# The Role of Inflammation, Oxidative Stress, Neuronal Damage, and Endothelial Dysfunction in the Neuropathology of Cognitive Complications in Diabetes: A Moderation and Mediation Analysis

**DOI:** 10.1002/brb3.70225

**Published:** 2025-01-19

**Authors:** Betul Sumbul‐Sekerci, Ozge Pasin, Ezgi Balkan, Abdusselam Sekerci

**Affiliations:** ^1^ Department of Clinical Pharmacy, Faculty of Pharmacy Bezmialem Vakıf University Istanbul Turkey; ^2^ Department of Biostatistics Faculty of Medicine Bezmialem Vakıf University Istanbul Turkey; ^3^ Department of Medical Biochemistry Bezmialem Vakif University Istanbul Turkey; ^4^ Health Sciences Institute Bezmialem Vakıf University Istanbul Turkey; ^5^ Department of Internal Medicine, Faculty of Medicine Bezmialem Vakif University Istanbul Turkey

**Keywords:** cognitive impairment, diabetes, endothelial dysfunction, inflammation, neuronal damage, oxidative stress

## Abstract

**Objective:**

Cognitive impairment is increasingly recognized as a complication of diabetes, yet the underlying pathology remains unclear. This study aims to investigate the roles of inflammation, oxidative stress, endothelial dysfunction, and neuronal damage in the neuropathology underlying diabetes related cognitive impairment.

**Methods:**

This study assessed 183 participants (54 prediabetes, 71 Type 2 diabetes mellitus [T2DM], and 58 controls) for cognitive performance using the Montreal Cognitive Assessment (MoCA). Blood samples were analyzed for interleukin‐6 (IL‐6), tumor necrosis factor‐alpha (TNF‐α), malondialdehyde (MDA), VCAM‐1/CD106, and neuron‐specific enolase (NSE) using ELISA. Mediation and moderator analysis methods were used to examine the roles of these biomarkers in diabetes‐related cognitive impairment.

**Results:**

After adjusting for age, education, and gender, group comparisons revealed significant cognitive impairment in patients with T2DM, particularly in visuospatial functions, naming, language, and memory performance, compared to the control group. The patients with T2DM and prediabetes exhibited similar performance in cognitive functions, except for language. Significant differences in VCAM‐1 and TNF‐α levels were observed; however, these biomarkers did not mediate the effect of T2DM and prediabetes on cognitive functions. Nevertheless, VCAM‐1 was found to moderate abstraction abilities in T2DM.

**Conclusion:**

Prediabetes represents a transitional stage not only for the pathology of diabetes but also for cognitive complications. Although there were correlations between cognitive performance and various cognitive scores, IL‐6, MDA, NSE, VCAM‐1, and TNF‐α did not play a mediator role in the neuropathology of diabetes‐related cognitive impairment.

## Graphical abstract

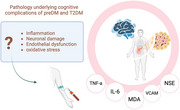

## 1 Introduction

In patients with Type 2 diabetes mellitus (T2DM), 53.2% exhibit microvascular and 27.2% exhibit macrovascular complications (Litwak et al. 2013). Managing these complications is crucial due to their significant impact on mortality and morbidity. Cognitive impairment, which can range from mild cognitive impairment (MCI) to dementia, is one of the less understood and less researched complications of T2DM, although it has garnered increasing attention recently (Hardigan, Ward, and Ergul 2016; Koekkoek et al. 2015).

Although studies have shown an increased risk of cognitive impairment and dementia in patients with T2DM (Alsharif et al. 2020; Ott et al. 1999; X. Zhang et al. 2019), the causal neuropathological relationship between diabetes and specific cerebral changes remains unclear (Biessels and Whitmer 2020). Furthermore, the effects of prediabetes—a transitional stage to T2DM—on cognitive functions and brain health are not well understood (Sundermann et al. 2021). It is crucial to elucidate the impact of diabetes‐related pathology on the brain, cognition, and neurodegenerative diseases (Beeri and Bendlin 2020).

Cognitive impairment in T2DM is thought to result from multiple etiologies, some specific to diabetes, and may be associated with complex pathologies and interindividual variations (Biessels and Despa 2018). Several pathways have been proposed to explain the relationship between diabetes and cognitive complications, including vascular system damage, protein misfolding, and inflammation or oxidation, which can promote neurodegenerative and vascular damage (Launer 2020). Diabetes‐induced endothelial dysfunction is considered a critical and initiating factor in the development of vascular complications (Shi and Vanhoutte 2017). The activation of the inflammatory cascade and endothelial dysfunction are significant in both the etiology of diabetes and the pathophysiological mechanisms of diabetes‐related complications (Goldberg 2009). Inflammation, by disrupting cerebral vasoregulation, has been highlighted as a major pathway that could trigger cognitive impairment in patients with T2DM (Chung et al. 2015). Increased levels of inflammatory cytokines have been associated with both the risk of T2DM and Alzheimer's disease (Liu et al. 2016; Tao et al. 2018). Additionally, proteins such as neuron‐specific enolase (NSE) have been shown to be useful in the early detection and monitoring of neurological complications like polyneuropathy, as NSE is released into endoneurial fluid and serum following neuronal damage (Elsharkawy et al. 2021; Li et al. 2013). Although numerous studies have investigated the relationship between these pathophysiological mechanisms and the pathology and various complications of diabetes, there is limited research examining their connection to cognitive impairment. Furthermore, studies specifically focusing on this relationship in prediabetes are lacking.

Our study aims to investigate the role of various biomarkers of inflammation, oxidative stress, vascular, and neuronal damage in diabetes‐related cognitive impairment in patients with prediabetes and T2DM, using advanced statistical analysis methods such as mediator and moderator analyses.

## 2 Methods

### 2.1 Participant

At a 95% confidence level and 80% power, when the effect size is evaluated according to the reference study (Zheng et al. 2019), a minimum of 16 participants per group is required for the study. Regarding the sample size suggested for structural equation modeling (SEM), it is commonly believed that there should be at least 10 cases for each measured variable. In this case, for a model with 5–10 variables, a sample size of around 150–200 participants is generally considered sufficient (Newsom 2018).

This cross‐sectional study was conducted with 183 patients (54 prediabetes, 71 T2DM, and 58 controls) who applied to the internal medicine outpatient clinic of Bezmialem Vakif University Faculty of Medicine Hospital between May 2023 and February 2024. The T2DM and preDM diagnosis of patients was evaluated according to ADA criteria (American Diabetes Association 2021), and their eligibility for the study based on inclusion or exclusion criteria was determined by an internal medicine specialist. Participants were included in the study if they were at least primary school graduates and aged between 30 and 65 years. Exclusion criteria included a history of significant psychiatric conditions, such as major depressive disorder or neurological disorders, such as dementia, cerebrovascular disease, intracranial infection, demyelinating disease, brain tumors, or head trauma. Participants receiving insulin therapy were excluded to avoid the confounding effects of hypoglycemia on cognitive function. Severe visual or auditory impairments that would hinder the administration of neuropsychological tests were also grounds for exclusion, as were conditions such as hypothyroidism and deficiencies in B12 or folic acid. Additionally, individuals with advanced chronic kidney disease (Stage 4 or 5), those experiencing significant fluctuations in blood glucose levels, and those with alcohol, substance, or drug dependency were excluded from the study. The patients/participants provided their written informed consent to participate in this study.

### 2.2 Cognitive Evaluation

The cognitive performance of the participants was assessed with the MoCA (Montreal Cognitive Assessment) test (Nasreddine et al. 2005). The test, which is a general screening test and lasts approximately 10 min, evaluates attention, visuospatial/executive functions, memory, language, abstraction, and orientation. The highest total score that can be obtained from the test is 30. According to Turkish validation studies, a score of 21 and above is considered normal, whereas a score below 21 is considered impairment in cognitive functions (Ozdilek and Kenangil 2014). Those with 12 years of education or less are corrected for education by giving one additional point.

### 2.3 Laboratory

Blood samples were collected from patients and the control group after 8 hours of fasting in the morning. The samples were placed in gel separation tubes, centrifuged at 3500 × *g* for 10 min at room temperature, and stored at −80°C until the study was completed. For the measurement of serum levels of interleukin‐6 (IL‐6), tumor necrosis factor‐alpha (TNF‐α), malondialdehyde (MDA), vascular cell adhesion molecule 1/cluster of differentiation 106 (VCAM‐1/CD106), and NSE using the sandwich ELISA method, the following kits were used: Elabscience Human IL‐6 ELISA Kit (Catalog No: E‐EL‐H6156), Elabscience Human TNF‐α ELISA Kit (Catalog No: E‐EL‐H0109), Elabscience MDA ELISA Kit (Catalog No: E‐EL‐0060), Elabscience VCAM‐1 CD106 (vascular cell adhesion molecule 1) ELISA Kit (Catalog No: E‐EL‐H5587), and Elabscience Human NSE ELISA Kit (Catalog No: E‐EL‐H104). All steps of the analysis were performed according to the protocol designed by the kit manufacturer (example protocol: https://www.elabscience.com/protocols‐elisa‐155.html). The routine clinical laboratory values, including fasting serum glucose, hemoglobin A1c, oral glucose tolerance test (OGTT), insulin, HOMA‐IR, LDL, HDL, triglycerides, TSH, vitamin B12, folic acid, and complete blood count, were retrospectively obtained from the Bezmialem Vakıf University Hospital's information system.

### 2.4 Statistical Analysis

Descriptive statistics for categorical variables in the study are presented as numbers and percentages, whereas descriptive statistics for quantitative variables are given as mean, standard deviation, median, minimum, and maximum. Relationships between categorical variables were examined using Pearson's chi‐square and Fisher Freeman Halton tests. The normality of the distribution of quantitative variables was assessed with the Shapiro–Wilk test. For the comparison of means between two independent groups with normally distributed quantitative variables, the independent samples *t*‐test (Student *t*‐test) was used. For non‐normally distributed quantitative variables, the Mann–Whitney *U* test was used to compare means between two independent groups. For comparisons of means between more than two independent groups with normally distributed variables, one‐way analysis of variance (ANOVA) was applied, and the LSD method was used for multiple comparisons. For non‐normally distributed variables, the Kruskal–Wallis test was used for comparisons of means between more than two independent groups, with the Dunn test used as a post hoc test. Relationships between quantitative variables were investigated using Pearson and Spearman correlation coefficients. To adjust for the effects of covariates and factors, ANCOVA analysis was performed, and means and standard deviations were provided with adjustments. When the dependent variable was quantitative, multivariate analyses used linear regression analysis; for binary dependent variables, binary logistic regression was employed; and for multinomial dependent variables, multinomial regression analysis was used. The stepwise method was used for variable selection in regression analyses. A statistical significance level of 0.05 was adopted, and calculations were performed using the SPSS software package (version 26, Chicago, IL, USA).

### 2.5 Mediation and Moderation Analysis

In mediation analyses, the presence of a third variable's effect between the independent and dependent variables is tested. This third variable explains how the independent variable affects the dependent variable. In the literature, this third variable is referred to as the mediator variable. This variable explains all or part of the effect of the independent variable on the dependent variable. The analysis is fundamentally based on the linear regression method. Hayes’ PROCESS macro was used for mediation analysis, employing the ordinary least‐squares framework to estimate direct and indirect effects. The significance of effects was evaluated using the Sobel test.

In moderation analysis, the effect of another variable on the relationship between the independent and dependent variables is examined. This variable influences the strength and direction of the relationship between the dependent and independent variables. Moderation analysis can be conducted using various statistical methods such as multiple regression, ANOVA, and SEM. Although a mediator variable explains the relationship between two variables, a moderator variable affects the strength and direction of this relationship. Moderator variables are key to understanding this context (Morrow, Duff, and Mayberry 2022). In this study, mediation effects were evaluated to investigate the relationships between dependent and independent variables. As the presence of these effects could not be detected, the potential for a moderating effect between the dependent and independent variables was assessed.

## 3 Results

The study was conducted with 183 participants, including 54 individuals with prediabetes, 71 with T2DM, and 58 controls. The education levels of the participants were distributed as follows: 89 completed primary school, 25 completed middle school, 44 completed high school, and 25 had a university degree or higher. There were 109 female participants (59.6%), with a mean age of 50.51 ± 7.29. Significant differences were found among the groups in terms of gender (*p* = 0.001), age (*p* = 0.001), and education level (*p* = 0.005). The mean age of the prediabetes group was 53.13 ± 6.38, the diabetes group was 50.49 ± 8.43, and the control group was 48.10 ± 5.66.

Clinical data of the patient groups were adjusted for age with nonparametric ANCOVA (QUADE'S) and compared in Table 1. There were significant differences between the groups in fasting glucose and HBa1C, as well as triglyceride, neutrophil, and lymphocyte levels.

**TABLE 1:** Clinical characteristics of the participants according to adjusted for age.

Variables	PreDM (*n* = 54)	T2DM (*n* = 71)	Control (*n* = 58)	*p*	Post hoc comparison
Fasting glucose (mg/dL)	105 ± 36.945 103 (86–217)	162.21 ± 36.216 148.5 (72–381)	90.82 ± 36.902 92 (80–99)	**<0.001**	1–2 ≤ **0.001** 1–3 ≤ **0.001** 2–3 ≤ **0.001**
HbA1c (%)	5.81 ± 1.108 5.75 (4.87–8.77)	7.67 ± 1.078 7.29 (5.21–13.20)	5.18 ± 1.106 5.20 (4.67–5.78)	**<0.001**	1–2 ≤ **0.001** 1–3 ≤ **0.001** 2–3 ≤ **0.001**
LDL (mg/dL)*	135.67 ± 41.197 134.6 (51–202)	138.89 ± 37.99 132.6 (63–389)	127.52 ± 41.158 130.65 (66.8–178)	0.270	
TSH (mIU/L)	2.11 ± 1.200 1.71 (0.13–5.84)	1.95 ± 1.220 1.71 (0.52–6.02)	1.88 ± 1.192 1.80 (0.33–5.21)	0.650	
Trigliserit (mg/dL)	145.18 ± 85.137 133 (44–387)	164.92 ± 78.216 142 (54–454)	131.13 ± 82.634 118 (38–405)	**0.041**	1–2 = 0.573 1–3 = 0.856 2–3 = **0.036**
B12 (ng/L)	343.55 ± 239.524 285 (168–2000)	330.71 ± 218.884 285 (154–899)	367.43 ± 228.489 331.5 (197–2000)	0.234	
Folic acid (µg/L)*	7.57 ± 8.822 7 (2.7–20.40)	8.05 ± 8.200 7.85 (2.5–17.30)	10.32 ± 8.623 8.7 (3.80–103)	0.196	
Neutrophil (10 × 3/µL)	4.31 ± 1.336 3.95 (2.41–7.39)	4.40 ± 1.285 4.25 (1.89–8.41)	3.68 ± 1.334 3.57 (1.59–8.85)	**0.003**	1–2 = 1.000 1–3 = **0.033** 2–3 = **0.003**
Lymphocyte (10 × 3/µL)*	2.56 ± 0.759 2.46 (1.18–4.41)	2.72 ± 0.730 2.64 (0.81–6.34)	2.30 ± 0.752 2.31 (0.92–4.20)	**0.006**	1–2 = 0.727 1–3 = 0.216 2–3 = **0.005**
Neutrophil/lymphocyte ratio	1.79 ± 0.798 1.717 (0.72–3.47)	1.74 ± 0.767 1.56 (0.83–6.14)	1.65 ± 0.790 1.50 (0–5.71)	0.208	
Hemoglobin (g/dL)*	13.65 ± 1.769 13.50 (10.74–16.20)	14.26 ± 1.685 14.32 (10.15–18.01)	13.71 ± 1.751 13.90 (8.68–17.40)	0.089	
Platelet (10 × 3/µL)*	258.43 ± 71.366 248.5 (138–497)	260.06 ± 67.981 250.50 (155–421)	256.84 ± 69.991 246 (87–546)	0.966	

*Note*: Variables with a normal distribution are indicated with (*). For variables that are normally distributed, an ANCOVA model was used by including covariates in the analysis. For variables without a normal distribution, nonparametric ANCOVA (QUADE's test) was applied. Post hoc comparisons were conducted using Bonferroni correction. Descriptive statistics are presented as mean ± standard deviation, median (minimum–maximum). Statistically significant values are highlighted in bold.

Abbreviation: T2DM, Type 2 diabetes mellitus.

The MoCA total score and cognitive domain subscores were compared between the groups, and the results are presented in Table 2. Significant differences were found between the groups in the MoCA total score and in all subdomains except for orientation. In the Model 2 adjusted for age, education, and gender covariates, the significance in attention and abstraction domains disappeared. However, according to the adjusted model, there was a significant impairment in total cognitive score in the T2DM group compared to the control and prediabetes groups (*p* < 0.001). Specifically, patients with T2DM showed significantly lower performance in visual‐spatial/executive function (*p* = 0.019), naming (*p* = 0.004), language (*p* = 0.017), and memory (*p* = 0.003) compared to the control group. Patients with T2DM also showed a significant decrease in performance only in the language domain compared to the prediabetes group (*p* = 0.02), whereas their performance in other cognitive subdomains was similar.

**TABLE 2:** Comparison of cognitive total score and subscores of the groups.

	Model 1					Model 2				
Variables	PreDM (*n* = 54)	T2DM (*n* = 71)	Control (*n* = 58)	*p*	Post hoc comparison	PreDM (*n* = 54)	T2DM (*n* = 71)	Control (*n* = 58)	*P*	Post hoc comparison
MoCA total score*	22.17 ± 3.947 22.5 (11–30)	21.66 ± 4.299 22 (11–30)	25.14 ± 3.006 26 (18–30)	**<0.001**	1–2 = 0.746 1–3 ≤ **0.001** 2–3 ≤ **0.001**	23.13 ± 3.50 22.5 (11–30)	21.81 ± 3.32 22 (11–30)	24.33 ± 3.53 26 (18–30)	**<0.001**	**1**–**2** = **0.036** 1–3 = 0.074 2–3 ≤ **0.001**
Visuospatial/executive	3.56 ± 1.040 4 (1–5)	3.61 ± 1.102 4 (1–5)	4.26 ± 1.069 5 (0–5)	**<0.001**	1–2 = 1.000 1–3 ≤ **0.001** 2–3 ≤ **0.001**	3.73 ± 1.07 4 (1–5)	3.64 ± 1.01 4 (1–5)	4.09 ± 1.08 5 (0–5)	**0.05**	1–2 = 0.623 1–3 = 0.082 2–3 = **0.019**
Naming	2.54 ± 0.503 3 (2–3)	2.49 ± 0.606 3 (1–3)	2.81 ± 0.395 3 (2–3)	**0.002**	1–2 = 0.919 1–3 = **0.004** 2–3 = **0.001**	2.61 ± 0.53 3 (2–3)	2.49 ± 0.50 3 (1–3)	2.77 ± 0.54 3 (2–3)	**0.016**	1–2 = 0.212 1–3 = 0.126 2–3 = **0.004**
Attention	4.39 ± 1.379 5 (1–6)	4.72 ± 1.514 5 (1–6)	5.10 ± 1.180 6 (2–6)	**0.009**	1–2 = 0.061 1–3 = **0.006** 2–3 = 0.171	4.80 ± 1.27 5 (1–6)	4.72 ± 1.21 5 (1–6)	4.95 ± 1.28 6 (2–6)	0.583	
Language	1.63 ± 1.051 1.50 (0–3)	1.32 ± 1.118 1 (0–3)	2.03 ± 0.878 2 (0–3)	**0.001**	1–2 = 0.126 1–3 = **0.043** 2–3 ≤ **0.001**	1.79 ± 1.00 1.50 (0–3)	1.37 ± 0.95 1 (0–3)	1.80 ± 1.01 2 (0–3)	**0.020**	**1**–**2** = **0.020** 1–3 = 0.953 2–3 = **0.017**
Abstraction	1.50 ± 0.607 2 (0–2)	1.31 ± 0.748 1 (0–2)	1.66 ± 0.548 2 (0–2)	**0.022**	1–2 = 0.198 1–3 = 0.175 2–3 = **0.006**	1.55 ± 0.66 2 (0–2)	1.33 ± 0.63 1 (0–2)	1.57 ± 0.67 2 (0–2)	0.081	
Memory	2.81 ± 1.375 3 (0–5)	2.44 ± 1.370 3 (0–5)	3.39 ± 1.346 4 (0–5)	**0.001**	1–2 = 0.147 1–3 = **0.025** 2–3 ≤ **0.001**	2.83 ± 1.44 3 (0–5)	2.47 ± 1.37 3 (0–5)	3.24 ± 1.46 4 (0–5)	**0.012**	1–2 = 0.162 1–3 = 0.138 2–3 = **0.003**
Orientation	5.74 ± 0.521 6 (4–6)	5.82 ± 0.425 6 (4–6)	5.91 ± 0.285 6 (5–6)	0.136		5.74 ± 0.429 6 (4–6)	5.82 ± 0.418 6 (4–6)	5.91 ± 0.432 6 (5–6)	0.234	

*Note*: Variables with a normal distribution are indicated with (*). For Model 1, ANOVA and Kruskal–Wallis tests were used. In Model 2, adjustments were made for age, education years, and gender. ANCOVA was applied for normally distributed variables by incorporating covariates into the model, whereas nonparametric ANCOVA (QUADE's test) was used for non‐normally distributed variables. Post hoc comparisons were conducted with Bonferroni correction. Descriptive statistics are presented as mean ± standard deviation, median (minimum–maximum). Statistically significant values are highlighted in bold.

Abbreviations: MoCA, Montreal Cognitive Assessment; T2DM, Type 2 diabetes mellitus.

Various serum biomarkers associated with inflammation, oxidative stress, and neuronal and vascular damage were compared between the groups, which were presented in Table 3. Significant differences were found, particularly in VCAM‐1 (*p* < 0.001) and TNF‐α (*p* = 0.010) levels. VCAM‐1 levels were significantly lower in patients with T2DM compared to both control (*p* < 0.001) and prediabetes (*p* = 0.004). Additionally, TNF‐α levels were significantly higher in patients with prediabetes compared to T2DM (*p* = 0.007).

**TABLE 3:** Comparison of various serum biomarkers between groups.

Serum biomarkers	PreDM (*n* = 54)	T2DM (*n* = 71)	Control (*n* = 58)	*p*	Post hoc comparison
VCAM‐1 (ng/mL)	71.75 ± 18.99 77.08 (27.02–110.34)	61.92 ± 18.479 63.18 (10.64–90.69)	75.99 ± 18.957 80.61 (20.35–101.48)	**<0.001**	1–2 = **0.004** 1–3 = 0.626 2–3 ≤ **0.001**
NSE (ng/mL)	4.07 ± 1.75 3.72 (2.54–10.30)	4.23 ± 1.710 3.73 (2.69–10.98)	4.26 ± 1.754 3.58 (2.55–12.78)	0.943	
IL‐6 (pg/mL)	1.91 ± 1.792 1.31 (0–16.58)	1.57 ± 1.752 1.29 (0.005–9.299)	1.28 ± 1.770 1.07 (0–5.642)	0.387	
MDA (ng/mL)	2450.26 ± 112.796 2479.65 (2143.40–2590.90)	2457.31 ± 109.761 2496.20 (2213.6–2594.5)	2486.02 ± 112.599 2510.70 (2194.20–2597.30)	0.288	
TNF‐α (pg/mL)	96.93 ± 65.254 86.73 (0–249.93)	59.78 ± 61.027 46.6 (0–209.68)	71.59 ± 62.577 62.83 (0–222.4)	**0.010**	1–2 = **0.007** 1–3 = 0.270 2–3 = 0.536

*Note*: In the analyses, the age variable was included as a covariate in the models. The effects of independent variables on groups were assessed using ANCOVA for normally distributed variables and nonparametric ANCOVA (QUADE's test) for non‐normally distributed variables. Post hoc comparisons were conducted using Bonferroni correction. Descriptive statistics are presented as mean ± standard deviation, median (minimum–maximum). Statistically significant values are highlighted in bold.

Abbreviations: IL‐6, interleukin‐6; MDA, malondialdehyde; NSE, neuron‐specific enolase; T2DM, Type 2 diabetes mellitus; TNF‐α, tumor necrosis factor‐alpha; VCAM, vascular cell adhesion molecule.

The relationship between cognitive performance and serum biomarkers was analyzed separately in the groups. In the prediabetes group, a significant relationship was found between VCAM‐1 and abstracting (*p* = 0.011, *r* = 0.342). In the T2DM group, significant correlations were observed between NSE and attention (*p* = 0.011, *r* = 0.30); MDA and MoCA total score (*p* = 0.050, *r* = −0.234), attention (*p* = 0.012, *r* = −0.297); IL‐6 and memory (*p* = 0.024, *r* = −0.271); TNF‐α and memory (*p* = 0.041, *r* = −0.246) (Table 4).

**TABLE 4:** Correlation between serum biomarkers and cognitive performance.

Group	Biomarker	Cognitive function	*p* value	Correlation (*r*)
PreDM	VCAM‐1	Abstracting	0.011	0.342
T2DM	NSE	Attention	0.011	0.300
	MDA	MoCA total score	0.050	−0.234
	MDA	Attention	0.012	−0.297
	IL‐6	Memory	0.024	−0.271
	TNF‐α	Memory	0.041	−0.246

Abbreviations: IL‐6, interleukin‐6; MDA, malondialdehyde; MoCA, Montreal Cognitive Assessment; NSE, neuron‐specific enolase; T2DM, Type 2 diabetes mellitus; TNF‐α, tumor necrosis factor‐alpha; VCAM‐1, vascular cell adhesion molecule 1.

**FIGURE 1:**
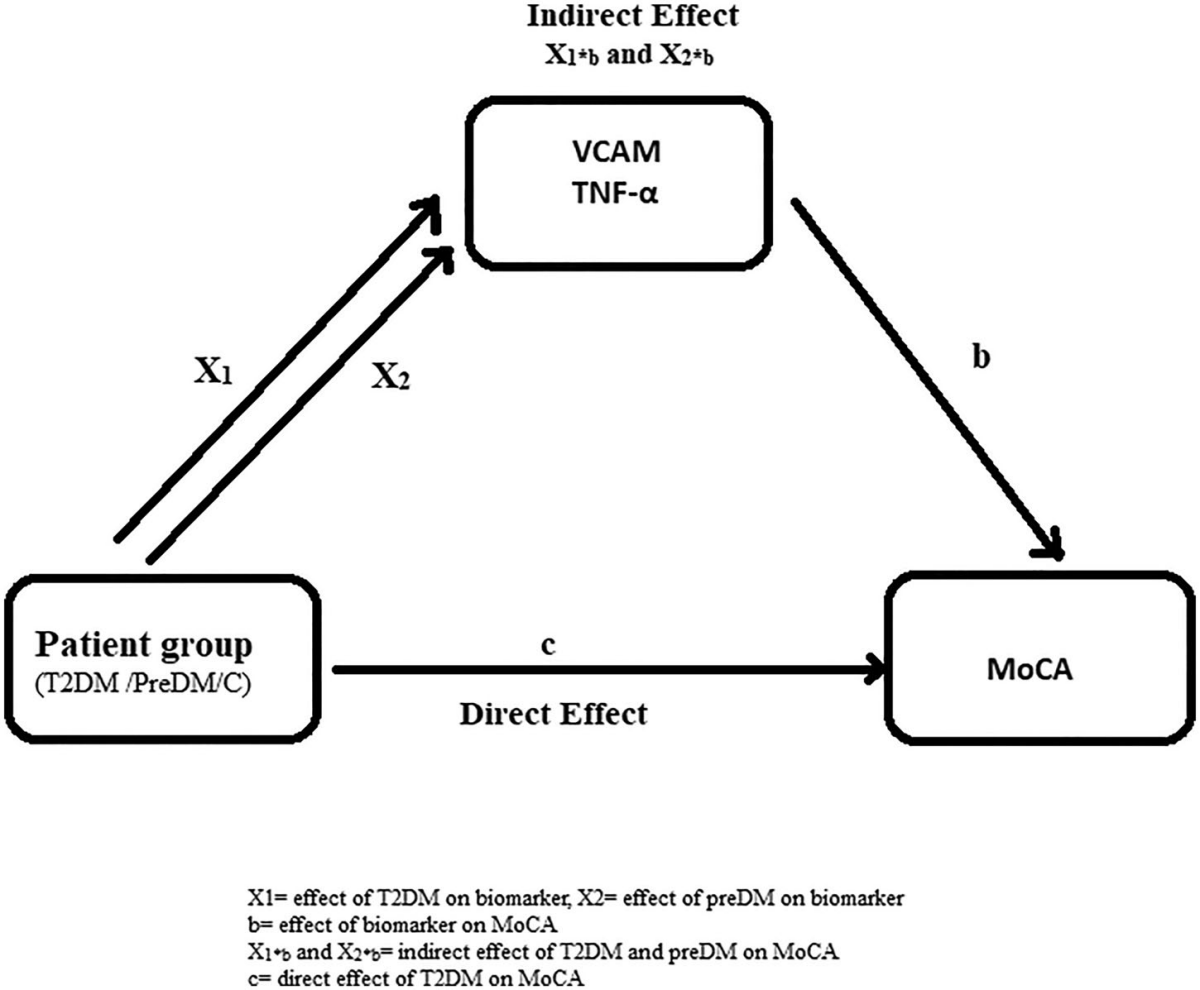
Mediator analysis of the effect of T2DM and prediabetes on cognitive functions through VCAM‐1 and TNF‐α levels. VCAM‐1: X_1b_ *p* = 0.394, Sobel test statistic value = 0.851; X_2b_ *p* = 0.818, Sobel test statistic value = 0.230. TNF‐α: X_1b_ *p* = 0.668, Sobel test statistic value = 0.428, X_2b_ *p* = 0.648, Sobel test statistic value = 0.456. MoCA, Montreal Cognitive Assessment; T2DM, Type 2 diabetes mellitus; TNF‐α, tumor necrosis factor‐alpha; VCAM‐1, vascular cell adhesion molecule 1.

Due to the significant differences in the levels of VCAM‐1 and TNF‐α among groups, the effect of VCAM‐1 and TNF‐α on cognitive impairment was examined through mediation analysis. The study had three subgroups (DM‐PreDM‐C). Dummy coding was used for the group variable to conduct the mediation analysis, with the control group taken as the reference group. Although the effect of T2DM was evaluated for X_1_ (T2DM&Control), the effect of preDM was evaluated for X_2_ (PreDM&Control). Sobel test was used to test the significance in the mediation analysis. For VCAM‐1, the indirect effect of T2DM on MoCA (X_1b_) was found to be *p* = 0.394, with a Sobel test statistic value of 0.851, whereas the indirect effect of prediabetes on MoCA (X_2b_) was *p* = 0.818, with a Sobel test statistic value of 0.230. In evaluating the mediator effect of TNF‐a, the indirect effect of T2DM on MoCA (X_1b_) was *p* = 0.668, with a test statistic of 0.428, and for the indirect effect of prediabetes on MoCA (X_2b_), it was *p* = 0.648, with a test statistic of 0.456. The analyses did not show an indirect effect on cognitive impairment associated with T2DM through VCAM‐1 and TNF‐α biomarkers. The mediator effect is presented in Figure 1.

**FIGURE 2:**
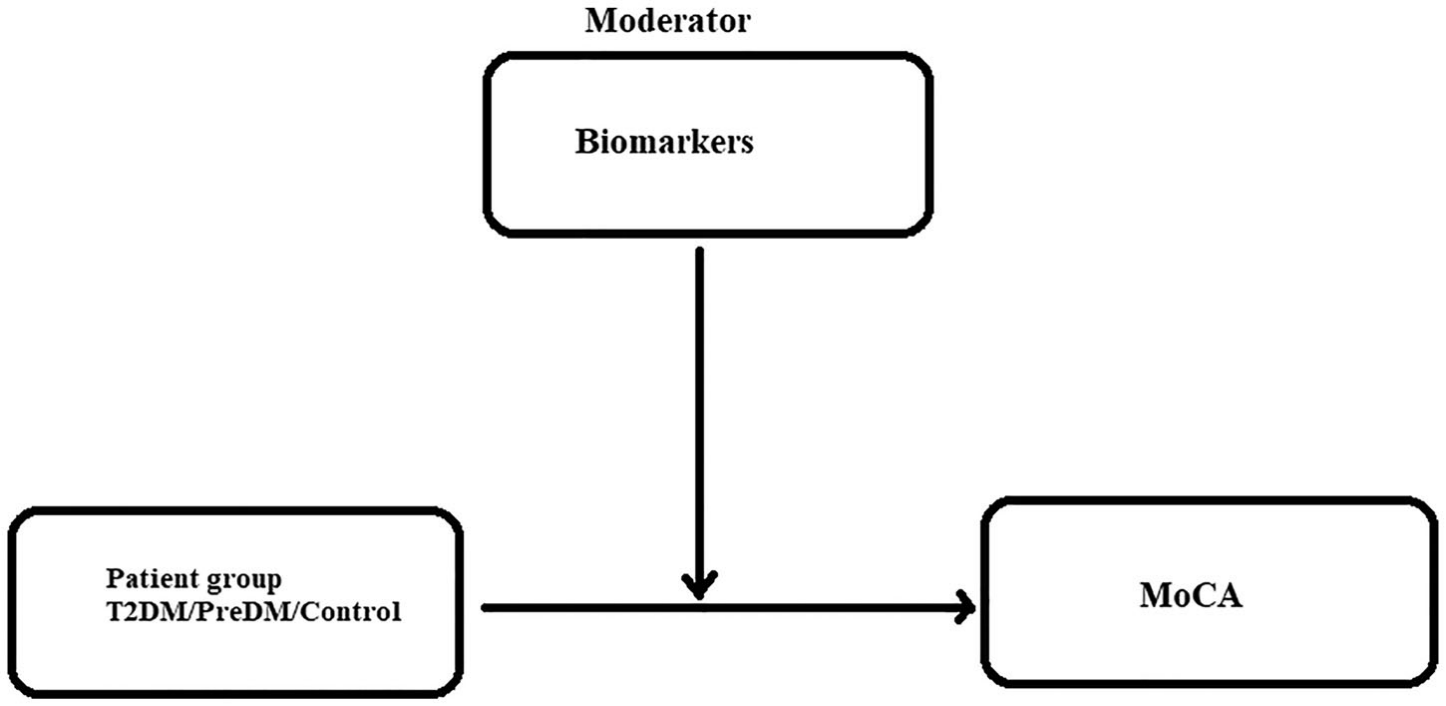
Moderator analysis of the effect of T2DM and prediabetes on cognitive functions through biomarkers. MoCA, Montreal Cognitive Assessment; T2DM, Type 2 diabetes mellitus.

The moderator effect is presented in Figure 2. In the moderator analysis, no moderating effect of the biomarkers NSE, IL‐6, and MDA on the impact of diabetes on cognitive scores was observed (*p* > 0.05). The role of VCAM‐1 and TNF‐α on total MoCA scores and subdomains was examined in Model 1, and after adjusting for age, in Model 2, as presented in Table 5. Accordingly, VCAM‐1 was found to have a moderating role on attention and abstraction functions in diabetes‐related cognitive impairment, with a moderating effect observed only in the abstraction domain when age was added as a covariate to the model (*p* = 0.040). Although TNF‐α was found to have a direct effect on naming functions (*p* = 0.040), it did not have a moderating function in diabetes‐related cognitive impairment (*p* = 0.43).

**TABLE 5:** Moderate effect of VCAM‐1 and TNFα on different cognitive performance.

		Model 1				Model 2			
Potential moderator	Cognitive score	Coefficient	*p*′	*R* ^2^ change	p*	Coefficient	*p*′	*R* ^2^ change	p*
VCAM‐1	MoCA total	0.022	0.375	0.023	0.089	−0.031	0.213	0.012	0.260
	Visual‐spatial	0.012	0.093	0.011	0.323	0.014	0.054	0.007	0.484
	Naming	0.003	0.321	0.024	0.095	0.004	0.242	0.017	0.180
	Attention	−0.002	0.756	0.031	**0.050**	0.001	0.978	0.021	0.133
	Language	0.009	0.166	0.022	0.110	0.012	0.085	0.014	0.243
	Abstraction	−0.001	0.842	0.036	**0.032**	−0.001	0.882	0.033	**0.040**
	Memory	0.002	0.799	0.001	0.868	0.003	0.715	0.001	0.906
	Orientation	−0.001	0.643	0.003	0.759	−0.001	0.628	0.003	0.787
TNF‐α	MoCA total	0.001	0.842	0.005	0.612	−0.001	0.887	0.005	0.616
	Visual‐spatial	−0.003	0.158	0.012	0.333	−0.004	0.146	0.013	0.311
	Naming	0.002	**0.040**	0.090	0.439	0.002	0.042	0.008	0.462
	Attention	0.001	0.806	0.004	0.670	0.001	0.854	0.005	0.604
	Language	−0.01	0.823	0.007	0.524	0.001	0.867	0.007	0.527
	Abstraction	0.001	0.352	0.004	0.666	0.001	0.361	0.004	0.678
	Memory	−0.001	0.831	0.007	0.495	−0.001	0.814	0.007	0.506
	Orientation	0.001	0.736	0.005	0.654	0.001	0.729	0.005	0.647

*Note*: In evaluating the moderating effect, Model 1 does not include age adjustment, whereas Model 2 adjusts for age. The table presents the coefficient values obtained for the variables in the models. *p*′ indicates the significance of the direct effect, and *p*∗ indicates the significance of the moderating effect. *R* ^2^ change represents the contribution of added variables to the explained variance. Statistically significant values are highlighted in bold.

Abbreviations: MoCA, Montreal Cognitive Assessment; TNF‐α, tumor necrosis factor‐alpha; VCAM‐1, vascular cell adhesion molecule 1.

When the moderator effect of VCAM‐1 is not taken into account, T2DM has no significant effect on abstraction (*B* = 0.001, *p* = 0.9929). However, considering the moderator effect of VCAM‐1, the impact of diabetes on cognitive functions is found to be significant. Accordingly, VCAM‐1 has a moderating role in diabetes‐related cognitive functions at low (*B* = −0.356, *p* = 0.0310 for 49.34 ng/mL), medium (*B* = −0.355, *p* = 0.003 for 69.28 ng/mL), and high (*B* = −0.354, *p* = 0.041 for 89.22 ng/mL) levels of VCAM‐1 values (*p* < 0.05). Prediabetes has a significant effect on abstraction (*B* = 0.0143, *p* = 0.0219). When examining the moderator role of VCAM‐1 in prediabetes‐related impairment of abstraction, a significant effect is observed at low levels (*B* = −0.502, *p* = 0.011 for 49.34 ng/mL), but the moderator effect disappears at medium (*B* = −0.216, *p* = 0.083 for 69.28 ng/mL) and high levels (*B* = 0.069, *p* = 0.653 for 89.22 ng/mL).

## 4 Discussion

In our study, we observed impaired cognitive performance in patients with T2DM and noted that prediabetes serves as a transitional stage not only for the pathology of diabetes but also for cognitive complications. The primary cognitive domains affected in diabetes‐related cognitive impairment were visuospatial functions, naming, language, and memory. Although there were correlations between cognitive performance and various cognitive scores, mediator analysis results indicated that the biomarkers IL‐6, MDA, NSE, VCAM‐1, and TNF‐α did not play a mediating role in the neuropathology of diabetes‐related cognitive impairment. However, VCAM‐1 demonstrated a moderator effect on abstraction functions.

Consistent evidence in the literature indicates that T2DM is an independent risk factor for cognitive dysfunction in the elderly (Dyer et al. 2021; Palta et al. 2017; Papunen et al. 2020). However, the literature on prediabetes is limited and inconsistent. Although a few studies have reported an association between prediabetes and cognitive impairment (Dybjer et al. 2018; Guo et al. 2020; Marseglia et al. 2019; Saçmaci et al. 2020), other studies have found no such relationship.(Cichosz, Jensen, and Hejlesen 2020; Papunen et al. 2020). Our study supports the existing literature highlighting cognitive complications in patients with T2DM. Although patients with prediabetes in our study exhibited lower performance compared to controls, the difference was not significant when compared to either the control or T2DM groups. We interpret this finding as indicative of prediabetes serving as a transitional phase for cognitive complications, similar to its role in the pathology of diabetes.

Our findings suggest that the profile of diabetes‐related cognitive impairment in our study is characterized primarily by deficits in visuospatial functions, naming, and memory, with language impairment being less prominent and areas such as attention, abstraction, and orientation being preserved. The literature shows limited studies investigating the phenotypes of cognitive impairment and underlying clinical correlations in patients with T2DM. The ENDBID study reported that T2DM is associated with lower memory scores (Dyer et al. 2021). Additionally, a meta‐analysis revealed that individuals with T2DM experience small to moderate declines in performance across all cognitive domains examined, including motor function, executive functions, processing speed, verbal and visual memory, and attention, compared to healthy controls (Palta et al. 2014). Although some studies in the literature suggest that memory impairment is more pronounced in diabetes‐related cognitive impairment (Roberts et al. 2014), others describe a cognitive profile marked by deficits in executive attention and other functions without amnestic changes (Yeung, Fischer, and Dixon 2009).

Diabetes‐associated microvascular damage and its potential effects on cognition are complex and multifactorial processes. Although the mechanisms are not fully understood, it is believed that oxidative stress and proinflammatory cytokines resulting from glucotoxicity may lead to low‐grade inflammation and endothelial dysfunction (Teodoro et al. 2018). Evidence suggests that in T2DM, vascular impairments due to hyperglycemia and chronic inflammation are associated with cognitive impairment and arteriosclerosis (Mehrabian et al. 2012; Novak et al. 2011). Furthermore, there is evidence that proteins such as NSE increase in neurological complications of diabetes, such as polyneuropathy, and that NSE is released into endoneurial fluid and serum following neuronal damage (Elsharkawy et al. 2021; Li et al. 2013). In our study, we found a significant relationship between NSE and attention, IL‐6 and memory, MDA and MoCA and attention, and TNF‐α and memory in patients with T2DM. In the prediabetes group, we observed a correlation between VCAM‐1 and abstraction. However, we did not find data supporting our hypothesis that these processes mediate cognitive complications in a complex neuropathological process based on causality.

Although various studies have investigated the levels of adhesion molecules in T2DM patients with microvascular complications such as neuropathy, retinopathy, and nephropathy, there are limited studies examining the cognitive complications of diabetes similar to our research. A meta‐analysis reported that IL‐6 and sVCAM‐1 levels are higher in T2DM patients with cognitive impairment, indicating an increased inflammatory‐vascular interaction associated with cognitive dysfunction in T2DM (Anita et al. 2022). One of the studies included in this meta‐analysis found that T2DM patients with Alzheimer's disease tend to have higher serum VCAM‐1 levels (L. Zhang and Mao 2020). Another study reported that T2DM patients with cognitive impairment had higher levels of sVCAM‐1 (Hosny et al. 2019). Additionally, elderly patients with MCI and T2DM, particularly those with comorbid depression, have been shown to have higher levels of soluble adhesion molecules and markers of low‐grade systemic inflammation (Gorska‐Ciebiada et al. 2015). However, our findings do not support these studies. In our research, we observed a significant decrease in VCAM‐1 and TNF‐α levels in T2DM, which might be attributed to the effects of antidiabetic medications on this complex pathology. The T2DM patients in our study were receiving various antihyperglycemic treatments in different groups and combinations. The significant drop in TNF‐α levels in T2DM, while they increased in patients with prediabetes, could be indicative of the influence of these treatments on the complex pathology associated with diabetes. Supporting this, various studies have shown that antihyperglycemic treatments such as metformin, GLP‐1 receptor agonists, and SGLT‐2 inhibitors are effective in reducing inflammation and biomarkers related to endothelial dysfunction (Garczorz et al. 2018; Luna‐Marco et al. 2023; Lund et al. 2008).

In our study, we explored whether the biomarkers had a moderator effect on diabetes‐related cognitive impairment, even if they did not have a direct mediator effect. The results of the moderator analysis indicated that NSE, IL‐6, MDA, and TNF‐α did not exhibit a moderating effect on diabetes‐related cognitive impairment. However, VCAM‐1 levels were found to have a moderator effect on abstraction in diabetes‐related cognitive impairment. Despite this, our study showed no significant impairment in the abstraction function associated with T2DM. This suggests that although VCAM‐1 levels may moderate the impact on abstraction, the effect is not substantial enough to result in clinically significant impairment in patients with diabetes.

Limitation of this study is that patients were not standardized according to their antidiabetic treatments. Antidiabetic treatments have the potential to influence both biomarker levels and cognitive performance. The impact of these treatments on cognitive functions remains a significant and ongoing debate in the literature. The effects of metformin on cognitive performance are inconclusive, with some studies reporting benefits and others finding no significant association (Lin et al. 2018; Newby et al. 2022; Pomilio et al. 2022). Notably, DPP‐4 inhibitors and GLP‐1 receptor agonists are consistently associated with cognitive improvements (Borzì et al. 2019; Meng et al. 2023; Watson et al. 2019). SGLT‐2 inhibitors have been suggested to positively impact cognitive functions (Kim et al. 2019; Mone et al. 2022; Mui et al. 2021), though clinical studies on SGLT‐2 inhibitors remain limited. Although some evidence highlights their potential cognitive benefits, including reduced risks of dementia and Parkinson's disease compared to DPP‐4 inhibitors, studies comparing SGLT‐2 inhibitors with incretin‐based therapies or metformin show mixed results (Mui et al. 2021). These findings underscore the need for further research to clarify the cognitive effects of different antidiabetic therapies. Therefore, optimizing and standardizing antidiabetic treatment strategies may be important for studies investigating cognitive functions and related biomarkers in patients with T2DM. Future studies that increase the sample size and group T2DM patients based on their treatments could provide more comprehensive insights.

## 5 Conclusion

Given the increasing incidence of T2DM and the aging population, the cognitive complications of diabetes are expected to become a more significant issue in the future. The evidence linking T2DM with cognitive impairment, coupled with the rapidly rising global prevalence of T2DM, underscores the importance of understanding the impact of diabetes‐related pathology on the brain, cognition, and neurodegenerative diseases. Identifying this relationship, especially at an early stage like prediabetes, could provide significant advantages in developing treatment and preventive strategies. Identifying risk factors and biomarkers for early diagnosis of diabetes‐related cognitive impairment is crucial. In addition to highlighting the importance of research in these areas, clinical guidelines should place greater emphasis on diabetes‐related cognitive impairment as a complication. Regular screening of patients with T2DM, and even initiating such screenings during the prediabetes stage, could be beneficial. Future research on the cognitive complications of diabetes and associated biomarkers can provide a broader understanding of the pathology and advance the early detection and treatment of these complications.

## Author Contributions

**Betul Sumbul‐Sekerci**: conceptualization, data curation, formal analysis, writing–original draft, writing–review and editing. **Ozge Pasin**: software, formal analysis, writing–review and editing, supervision. **Ezgi Balkan**: investigation, writing–review and editing. **Abdusselam Sekerci**: conceptualization, data curation, writing–review and editing.

## Ethics Statement

The studies involving human participants were reviewed and approved by Bezmialem Vakif University Clinical Research Ethics Committee (08.02.2023‐3/7).

## Conflicts of Interest

The authors declare no conflicts of interest.

### Peer Review

The peer review history for this article is available at https://publons.com/publon/10.1002/brb3.70225

## Data Availability

The data that support the findings of this study are available from the corresponding author upon reasonable request.
